# Imaging oxytocin × dopamine interactions: an epistasis effect of CD38 and COMT gene variants influences the impact of oxytocin on amygdala activation to social stimuli

**DOI:** 10.3389/fnins.2013.00045

**Published:** 2013-04-01

**Authors:** Carina Sauer, Christian Montag, Martin Reuter, Peter Kirsch

**Affiliations:** ^1^Department of Clinical Psychology, Central Institute of Mental Health Mannheim and Medical Faculty Mannheim, University of HeidelbergMannheim, Germany; ^2^Department of Personality and Biological Psychology, University of BonnBonn, Germany; ^3^Center for Economics and Neuroscience, University of BonnBonn, Germany

**Keywords:** oxytocin, dopamine, CD38, COMT, amygdala, fMRI

## Abstract

Although oxytocin (OT) has become a major target for the investigation of positive social processes, it can be assumed that it exerts its effects in concert with other neurotransmitters. One candidate for such an interaction is dopamine (DA). For both systems, genetic variants have been identified that influence the availability of the particular substance. A variant of the gene coding for the transmembrane protein CD38 (rs3796863), which is engaged in OT secretion, has been associated with OT plasma level. The common catechol-O-methyltransferase (COMT) val158met polymorphism is known to influence COMT activity and therefore the degradation of DA. The present study aimed to investigate OT × DA interactions in the context of an OT challenge study. Hence, we tested the influence of the above mentioned genetic variants and their interaction on the activation of different brain regions (amygdala, VTA, ventral striatum and fusiform gyrus) during the presentation of social stimuli. In a pharmacological cross-over design 55 participants were investigated under OT and placebo (PLA) by means of fMRI. Brain imaging results revealed no significant effects for VTA or ventral striatum. Regarding the fusiform gyrus, we could not find any effects apart from those already described in Sauer et al. (2012). Analyses of amygdala activation resulted in no gene main effect, no gene × substance interaction but a significant gene × gene × substance interaction. While under PLA the effect of CD38 on bilateral amygdala activation to social stimuli was modulated by the COMT genotype, no such epistasis effect was found under OT. Our results provide evidence for an OT × DA interaction during responses to social stimuli. We postulate that the effect of central OT secretion on amygdala response is modulated by the availability of DA. Therefore, for an understanding of the effect of social hormones on social behavior, interactions of OT with other transmitter systems have to be taken into account.

## Introduction

In social neuroscience, the neuropeptide oxytocin (OT) has become a major target for the investigation of positive social processes (Meyer-Lindenberg et al., 2011). However, although it can be shown that OT enhances positive social behavior (Yamasue et al., 2012), it must be assumed that the neuropeptide exerts its effects in concert with other neurotransmitters. One of the main candidates for such an interaction is dopamine (DA) (Skuse and Gallagher, 2009). Interactions between the OT and the DA system have been investigated for a longer time in animal research, mainly in the context of bonding and sexual behavior (Liu and Wang, 2003). While OT is strongly involved in establishing pair bonding, DA is thought to make mating a rewarding experience (Young and Wang, 2004).

Interconnections between both systems on the molecular und structural level probably build the fundament for these behavioral effects. DA receptors can be found on OT neurons in various brain regions particularly in the nuclei of the hypothalamus, where the OT system has its seeds (Baskerville et al., 2009). Furthermore, OT and DA receptors are collocated in regions of the mesolimbic DA system (Insel and Shapiro, 1992) and the injection of OT in a core region of this system, the ventral tegmental area (VTA) increases DA release in the nucleus accumbens, another core region of the mesolimbic DA system which receives projections from the VTA (Melis et al., 2007). Very recently the existence of D2-OT-receptor heteromers in the rat striatum has been proposed which might constitute a molecular mechanism underlying these OT-DA interactions (Romero-Fernandez et al., 2012).

In contrast to this extended animal literature on the interplay between OT and DA, much less is known about these interactions in humans. Very recently Love and colleagues found an effect of a common gene polymorphism (rs4813625) on the oxytocin receptor gene (OXTR) on the dopaminergic response to stress (Love et al., 2012). In a [11C] raclopride positron emission tomography study, they found an increased DA release in the ventromedial caudate during pain induced stress in female but not male carriers of the rs4813625 C-allele. They interpret their result as reflecting the impact of the oxytocin receptor on stress induced DA release.

In human neuroimaging research, the impact of OT on brain functions has mainly been investigated in OT challenge studies looking on the effect of intranasal OT application on neurobiological correlates of social cognitive processes (Zink and Meyer-Lindenberg, 2012). The most robust result is an attenuation of amygdala activation to social stimuli under OT compared to placebo, which was already demonstrated in our first study on that issue (Kirsch et al., 2005) and which could be replicated in several studies using different stimulation paradigms (e.g., Domes et al., 2007; Baumgartner et al., 2008; Petrovic et al., 2008). However, the amygdala is not only a mediator of OT effects, but is also an important target of the DA system. In humans, a modulatory effect of DA on amygdala activity during processing of negative emotional pictures (Kienast et al., 2008) and faces (Tessitore et al., 2002) has been shown. Furthermore, dopaminergic neurons in the central amygdala are activated during fear learning (Guarraci et al., 1999), potentially by a DA mediated attenuation of inhibitory signaling in the amygdala (Naylor et al., 2010). The amygdala might therefore also be an interesting region of interest (ROI) for the investigation of OT-DA interactions. Interestingly, paralleling their former results on OT injection in the VTA (Melis et al., 2007), Melis and colleagues also found an increase of mesolimbic DA release after OT injection into the amygdala (Melis et al., 2009).

To investigate OT × DA interactions in humans, an appropriate approach could be to look for epistasis effects between genetic variants that influence one or both of the systems. For both systems, OT and DA, functional genetic variants have been identified that influence the secretion or availability of the particular substance. For the OT system, the transmembrane protein CD38 has recently gained lots of attention. Although CD38 is expressed on different types of cells and is mainly known for its role in the immune system, it was recently found to play an important role in central OT secretion and to modulate social behavior (Jin et al., 2007). Importantly, the effect of CD38 on transmitter secretion was found to be specific to OT while the secretion of other transmitters, like striatal DA, or hypothalamic vasopressin was not affected. CD38 knockout mice show reduced OT levels and impaired social behavior. In humans, a common single nucleotide polymorphism (SNP) has been identified on the CD38 gene (rs3796863) that is associated with reduced CD38 expression in lymphoblasts (Lerer et al., 2010), reduced OT plasma levels and parental touch (Feldman et al., 2012) and also with autism spectrum disorder, a condition associated with severe social deficits (Lerer et al., 2010; Munesue et al., 2010). The presence of the C allele which was found to be associated with autism results in a reduced CD38 expression. Furthermore, we could recently show that this genetic variant also modulates the brain response to social stimuli in the fusiform gyrus, an important node of the social brain (Sauer et al., 2012). Here, the C allele was associated with stronger activation of the fusiform area.

For DA, the enzyme catechol-O-methyltransferase (COMT) is involved in the extraneuronal degradation of the transmitter and therefore influences its availability in the brain. A common variant on the gene coding for the enzyme, the val158met SNP (rs4860), is known to strongly influence the enzymatic activity of COMT (Chen et al., 2004). Carriers of the met allele show reduced enzymatic activity and therefore reduced degradation of the transmitter. On the brain level, this genetic variant has been shown to modulate brain responses during both, executive cognition and emotional processing paradigms and there is evidence for a pleiotropic action of the gene with the met allele favoring executive cognition and the val allele favoring emotional processing (Mier et al., 2010). Goldman and colleagues postulate a warrier/worrier dichotomy with the val allele supporting a stress resistant but slightly cognitively restricted and the met allele a cognitively superior but affectively more labile phenotype (Goldman et al., 2005). This dichotomy explaining the persistence of both alleles is supported by data demonstrating increased pain stress tolerance in val allele and an increased affective response to pain in met allele carriers (Zubieta et al., 2003). Interestingly, very recently this model was further supported by data showing that the superiority of met allele carriers in executive cognitive functions is diminished under social stress (Buckert et al., 2012).

While an impact of the CD38 gene polymorphism rs3796863 on amygdala could not be demonstrated so far (Sauer et al., 2012), a number of studies investigated effects of the COMT genotype on amygdala responses (Smolka et al., 2005, 2007; Drabant et al., 2006; Domschke et al., 2008, 2012; Kempton et al., 2009; Rasch et al., 2010; Williams et al., 2010; Lelli-Chiesa et al., 2011; Lonsdorf et al., 2011). However, these studies produced very inconsistent results ranging from no effect of rs4860, increased activation in val allele carriers or in met allele carriers to genotype × gender interactions. Therefore, it can be assumed that the effect of the particular genetic variant is modulated by other genes in terms of epistasis effects. Given an OT-DA interaction, the effect of the COMT genotype could be modulated by the CD38 genotype.

This study was conducted to explore COMT × CD38 genotype interactions in the human brain. Therefore, we further analyzed the data from our recently published OT challenge study (Sauer et al., 2012). Within this dataset, we investigated the influence of the polymorphisms rs379686 and rs4860 and their interaction on four different brain regions during the presentation of socially relevant stimuli. First, we focused on the amygdala because of its central role in human OT research and the heterogeneous results for COMT genotype. Second, given the results from animal research, we were interested in dopaminergic structures like the VTA and the ventral striatum. Third, for the sake of completeness, we also included the fusiform gyrus since it was mainly influenced by CD38 genotype in our previous study (Sauer et al., 2012). Furthermore, to test the impact of exogenous OT on potential genetic effects, we applied a pharmacological cross-over design where participants were investigated under OT and placebo (PLA).

## Materials and methods

### Participants

The sample was already described elsewhere (Sauer et al., 2012). It consists of 55 healthy young men of European ancestry (*M* = 24.9 ± 2.6 years). Only participants with no history of psychiatric or neurological diseases were included. All had at least 12 years of education, were non-smokers or smoked only occasionally and, except for one, all were right-handed. With respect to the CD38 SNP (rs3796863), 30 were homozygotic carriers of the C allele (CC), 23 were heterozygotes (CA) and 2 were homozygotic carriers of the A allele (AA). For subsequent statistical analyses, we pooled together AA and CA genotypes (A+) and compared them to the CC carriers (A−). Recent studies revealed the A− variant being associated with lower plasma OT levels and a higher risk for autism-spectrum-disorders compared to the A+ variant (Lerer et al., 2010; Munesue et al., 2010; Feldman et al., 2012).

With respect to the COMT val158met SNP (rs4680), 11 subjects were homozygotic carriers of the met allele (met/met), 31 were heterozygotes (val/met) and 13 were homozygotic carriers of the val allele (val/val) which has been shown to lead to a higher COMT activity and lower DA levels in prefrontal cortex (Chen et al., 2004). The distribution of genotype combinations is presented in Table 1. All genotype distributions are in Hardy-Weinberg-Equilibrium (COMT Val158Met: *Chi*^2^ = 0.91, *df* = 1, n.s.; CD38 (rs3796863): *Chi*^2^ = 0.91, *df* = 1, n. s.).

**Table 1:** **Distribution of CD38 and COMT genotypes in the sample**.

		**COMT (val158met polymorphism)**			**Total**
		**val/val**	**val/met**	**met/met**	
**CD38 (rs3796863)**	**A+**	5	14	6	25
	**A−**	8	17	5	30
	**Total**	13	31	11	55

Between the groups, there were no differences in age, education or order of substance application (all *p* > 0.1).

The study was approved by the ethics board of the German Psychological Society (DGPs) and all participants gave written informed consent.

### Experimental design and procedure

The experimental design and procedures are described in detail elsewhere (Sauer et al., 2012). We performed a double-blind placebo-controlled cross-over study. All participants attended two fMRI sessions at intervals of 1 week. They were instructed to abstain from alcohol and nicotine for 12 h before the session and from caffeine for 3 h. In each session, participants administered themselves either placebo or a dose of 25 IU of OT (Syntocinon Spray, Novartis, Austria; 5 puffs alternating nostrils, each with 5 IU) intranasally under the supervision of the investigator. To reach a sufficient and stable level of the substance in the brain (Born et al., 2002), the application took place about 30 min prior to the start of the fMRI experiment. In the meantime, participants completed several questionnaires to control for substance effects on different variables like mood, arousal etc.

Each MRI session consisted of a 5 min anatomical scan and two different fMRI paradigms. The anatomical scan was performed during the last 5 min of the 30 min time gap between substance application and the first fMRI experiment. Both fMRI experiments were designed to investigate aspects of social cognition. The first one was an extended version of the Hariri face matching task which is known to robustly activate the amygdala and other parts of the social brain (Hariri et al., 2002a,b, 2003). The task is implemented as a block design and requires subjects to match one of two presented stimuli to a simultaneously presented target stimulus. In the extended version we had five different conditions which were repeatedly presented in a non-randomized order. Four conditions consisted of pictures of emotional faces from the Pictures of Facial Affect series (Ekman and Friesen, 1976), or socially relevant scenes from the International Affective Picture System (Lang et al., 2008) with either positive or negative valence. In addition, geometrical shapes were presented in a control condition. Each social emotional condition was presented four times in 30 s blocks consisting of six trials per block. After each social emotional condition, the control condition was presented in 15 s blocks à six trials. The second fMRI paradigm was on gaze processing. However, as we do not further refer to this paradigm here, we won't describe it in detail.

### fMRI data acquisition

fMRI data were acquired on a 3T Siemens TRIO scanner (Siemens Medical Systems, Erlangen, Germany) with the following parameters for the functional MRI scans using EPI sequences: 30 axial slices à 4 mm, 1 mm gap, TR = 2 s, TE = 30 ms, FoV 192 × 192 mm, flip angle 80°. Anatomical data were obtained from a T1 weighted three dimensional MPRAGE sequence (192 sagittal slices of 1 mm thickness, TR = 2.3 s, TE = 3.03 ms, FoV 256 × 256 mm, flip angle 9°).

### fMRI data analysis

fMRI data were analyzed using SPM8 (http://www.fil.ion.ucl.ac.uk/spm). Preprocessing procedures included realignment to the first image, slice-time correction, spatial normalization into a standard stereotactic space with a voxel size of 2 × 2 × 2 mm using the Montreal Neurological Institute (MNI) template and smoothing with an 8 mm full width at half maximum (FWHM) Gaussian kernel.

After preprocessing, a general linear model (GLM) incorporating both substance conditions (PLA, OT) as separate sessions was applied for each subject. For each session, five task regressors (one for each experimental condition) and six motion parameters were included. On the first level we specified contrasts for both substance conditions separately (OT, PLA) and for the comparison between substances (OT vs. PLA).

Since we were interested in general aspects of social vs. non-social processing, we defined a contrast comparing all social stimuli to the control stimuli (contrast “social > non-social”). We also defined contrasts comparing each social condition separately to the control condition (“negative faces > non-social”, “positive faces > non-social”, “negative scenes > non-social”, “positive scenes > non-social”). However, these contrasts did not reveal any additional aspects but the same results as the combined contrast. Therefore, we decided to only report results for the combined contrast since the single contrasts have a lower power to detect subtle gene effects and show only marginally significant results.

Finally, we defined an interaction contrast by combining the task conditions with the substance conditions (social—non-social condition under PLA > social—non-social condition under OT).

On the second level, we performed multiple regression analyses for each first level contrast using the information of both polymorphisms independently and their interaction term as regressors. We also added substance order as a covariate of no interest to control for potential influences. To particularly address genetic effects on amygdala, VTA, ventral striatum and fusiform gyrus, we conducted ROI analyses. For the amygdala and the fusiform gyrus, we used anatomically defined Anatomical Automatic Labeling (AAL) masks provided by the Wake Forest University (WFU) PickAtlas software (http://fmri.wfubmc.edu). Masks for VTA and ventral striatum were created with the MARINA software tool (Walter et al., 2003). For each ROI analysis independently, we applied a significance level of *p* < 0.05 corrected for multiple comparisons using Family Wise Error (FWE) correction and an additional cluster size threshold of *k* = 10 contiguously activated voxels.

To further explore results from CD38 × COMT genotype × substance interactions on amygdala activation in detail, we first identified the peak voxel from the CD38 × COMT interaction effect on the contrast (social—non-social condition under PLA > social—non-social condition under OT) for left and right amygdala separately. We then extracted mean parameter estimates from that voxels + 5 mm sphere for the PLA and OT conditions separately. Afterwards, we further analyzed these data using GLM repeated measures procedures implemented in IBM SPSS 20 (IBM Inc., Armonk, NY.). Models consisted of valence (positive vs. negative) and substance condition (OT vs. PLA) as within-subject factors, CD38 and COMT genotypes as between-subject factors and substance order as a covariate of no interest.

### Analysis of behavioral data

For the analysis of behavioral effects, we analyzed the median of the response time (RT) for each condition. To control for baseline effects, we then computed differences between the social and the non-social conditions for both valences separately. These difference RTs were then incorporated into a GLM by means of IBM SPSS 20. Paralleling the analyses of amygdala activation, the GLM comprised valence and substance as within-subject factors, CD38 and COMT genotype as between-subject factors and substance order as covariate of no interest.

### Genotyping procedures

DNA was extracted from buccal cells to avoid a selective exclusion of subjects with blood and injection phobia. Automated purification of genomic DNA was conducted by means of the MagNA Pure® LC system using a commercial extraction kit (MagNA Pure LC DNA isolation kit; Roche Diagnostics, Mannheim, Germany). Genotyping was performed by real time-polymerase chain reaction (RT-PCR) using fluorescence melting curve detection analysis by means of the Light Cycler System (Roche Diagnostics, Mannheim, Germany). The primers and hybridization probes used (TIB MOLBIOL, Berlin, Germany) were as follows:

For CD38: forward primer: 5′-ACACTGAAGAAACTTGTCAGGTCTA-3′; reverse primer: 5′-CTTGGTTGCTGCTCCTACTGTT-3′; sensor hybridization probe: 5′-TTTGACCATCAGGTGGCA–FL -fluorescein-3′: anchor hybridization probe: 5′-LCRed640-GGATAGCTCCCCTCCCGACA-phosphate-3′.

For COMT: forward primer: 5′-GGGCCTACTGTGGCTACTCA-3′; reverse primer: 5′-GGCCCTTTTTCCAGGTCTG-3′; anchor hybridization probe: 5′-LCRed640-TGTGCATGCCTGACCCGTTGTCA-phosphate-3′; sensor hybridization probe: 5′-ATTTCGCTGGCATGAAGGACAAG -fluorescein-3′.

PCR runs comprised 55 cycles of denaturation (95°C, 0 s, ramp rate 20°C s-1), annealing (57°C, 10 s, ramp rate 20°C s-1) and extension (72°C, 10 s, ramp rate 20°C s-1) which followed an incubation period of 10 min to activate the FastStart Taq DNA Polymerase of the reaction mix (Light Cycler FastStart DNA Master Hybridization Probes, Roche Diagnostics, Mannheim, Germany). After amplification a melting curve was generated by holding the reaction time at 40°C for 2 min and then heating slowly to 95°C with a ramp rate of 0.2°C s-1. The fluorescence signal was plotted against temperature to yield the respective melting points (Tm) of the two alleles for both SNPs respectively. Tm for the CD38 C allele was 54.2°C and 61.5°C for the A allele. Tm for the COMT val allele was 59.00°C and 64.50°C for the met allele.

## Results

### fMRI results

**Figure 1:**
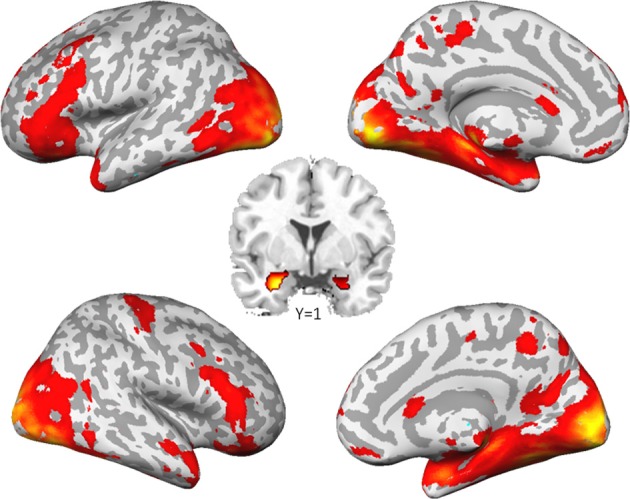
**Brain activation to social stimuli compared to non-social stimuli.** (*p* < 0.05, family wise error corrected for the whole brain).

As already reported in Sauer et al. (2012), the comparison of social to control stimuli led to a largely spread cortical and sub-cortical brain activation (Figure 1). As expected, we found a strong activation of the bilateral amygdala (left amygdala: cluster-size = 132 voxels, peak voxel (*x* = −22, *y* = −5, *z* = −20): *T*_(53)_ = 12.68, *p*_FWE_ < 0.001; right amygdala: cluster-size = 202 voxels, peak voxel (*x* = 26, *y* = 1, *z* = −26): *T*_(53)_ = 17.57, *p*_FWE_ < 0.001).

ROI analyses of VTA and ventral striatum revealed no significant results, neither for the single SNPs, nor for their interaction. Regarding fusiform gyrus activation, we found no significant results in addition to those already reported in Sauer et al. (2012).

**Figure 2:**
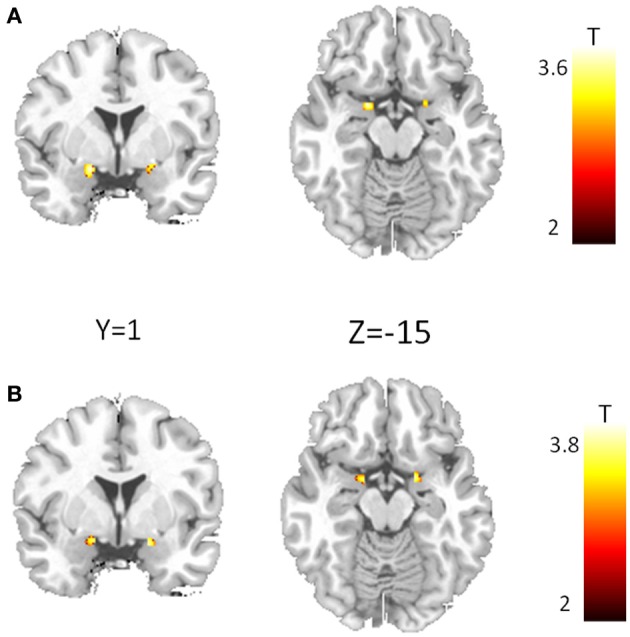
**Epistasis effect between CD38 and COMT in the amygdala. (A)** Voxel showing a significant three-way interaction between CD38, COMT, and substance. **(B)** Interaction between CD38 and COMT for the placebo condition. (*p* < 0.05, FWE corrected for the region of interest).

Analyses of amygdala activation revealed no gene main effect, neither for CD38 nor for COMT. Furthermore, there was no substance main effect and no gene × substance interaction for any variant. However, we found a significant gene × gene × substance interaction (Figure 2A) in the bilateral amygdala (left amygdala: cluster-size = 42 voxels, peak voxel (*x* = −18, *y* = −1, *z* = −12): *T*_(50)_ = 3.54, *p*_FWE(ROI)_ = 0.01; right amygdala: cluster-size = 21 voxels, peak voxel (*x* = 20, *y* = −1, *z* = −10): *T*_(50)_ = 3.25, *p*_FWE(ROI)_ < 0.05). This interaction is exclusively driven by a significant gene × gene interaction occurring under PLA (Figure 2B; left amygdala: cluster-size = 25 voxels, peak voxel (*x* = −14, *y* = −3, *z* = −12): *T*_(50)_ = 3.8, *p*_FWE(ROI)_ < 0.01; right amygdala: cluster-size = 23 voxels, peak voxel (*x* = 18, *y* = −1, *z* = −15): *T*_(50)_ = 3.68, *p*_FWE(ROI)_ < 0.01).

**Figure 3:**
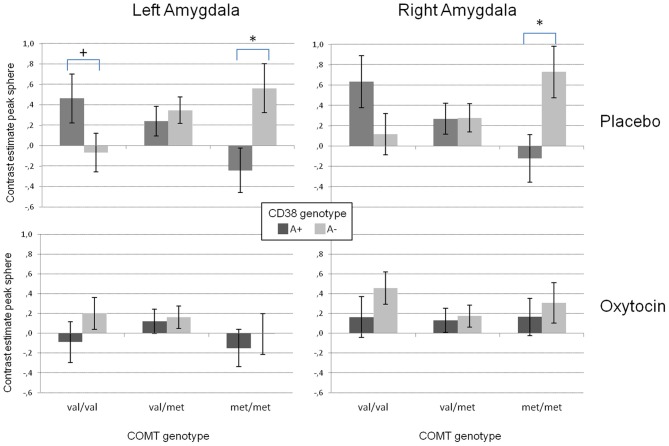
**Mean contrast estimates for the region of interest (amygdala peak voxel from the SPM model + 5 mm sphere) for the contrast “social > non-social” in the left and right amygdala reflecting the three-way interaction between CD38, COMT, and the substance.** While there is a clear interaction between the investigated genetic variants in the placebo condition (**upper panels**), this interaction disappears for the oxytocin condition (**lower panels**). (^+^*p* < 0.1, ^*^p < 0.05).

Our analyses on the extracted data from the peak voxel sphere replicated the three-way interaction between substance and the two genotype groups on both sides (left amygdala: *F*_(2/48)_ = 5.42, *p* < 0.01; right amygdala: *F*_(2/48)_ = 4.53, *p* < 0.02). As displayed in Figure 3, there was a strong modulation of the effect of CD38 on bilateral amygdala activation to social stimuli by the COMT genotype. While A+ carriers showed strongest amygdala activation when they were homozygote val allele carriers and lowest activation when they were homozygote met allele carriers, the opposite pattern was observed for A− carriers. In contrast, under OT this epistasis effect was completely diminished resulting in no differences between the different genotype configurations.

**Figure 4:**
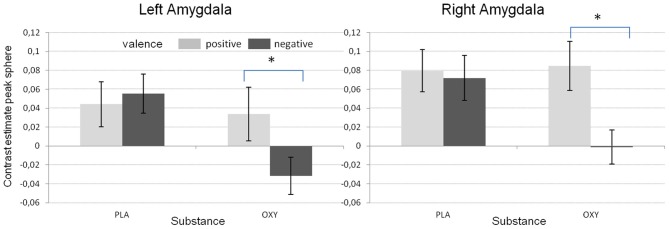
Mean contrast estimates for the different valence conditions and the two substances reflecting the significant interaction between valence and substance (^*^p < 0.05).

In addition, the GLM revealed a significant substance effect for the left amygdala [*F*_(1/48)_ = 14.02, *p* < 0.001). As can be seen from Figure 3, and as reported before (e.g., Kirsch et al., 2005), the activation in the left amygdala was reduced under OT when compared to PLA. However, this effect was not observed for the right amygdala which might be due to a significant valence × substance interaction reflecting that the OT attenuation effect was only present for the negative but not the positive valent stimuli [left amygdala: *F*_(1/48)_ = 19.59, *p* < 0.001; right amygdala: *F*_(1/48)_ = 5.31, *p* < 0.05, see Figure 4].

### Behavioral results

Analysis of the RTs revealed a significant valence main effect [*F*_(1/48)_ = 7.53, *p* < 0.01] with reduced RTs during negative when compared to positive stimuli. In addition we found a significant substance × CD38 interaction [*F*_(1/48)_ = 4.73, *p* < 0.05] reflecting reduced RTs under OT specifically in the A− group. This effect was already reported in our previous publication (Sauer et al., 2012).

## Discussion

In the present study, we used an exploratory approach to test for OT and DA associated gene effects and gene × gene interactions on brain response to social stimuli as well as the modulation of this response by OT. First of all, as already reported in Sauer et al. (2012), presenting social stimuli of positive and negative valence activated the bilateral amygdala allowing us to test for genetic effects on this phenotype. Furthermore, at least with respect to the analysis on the extracted sphere data, we replicated the often shown amygdala dampening effect of OT. Interestingly, in contrast to other reports (Domes et al., 2007) the amygdala inhibition in the present study was exclusively observed during negative valent stimuli (Figure 4) supporting the idea of an anxiety or social stress reducing effect of OT.

Regarding our main focus on the four different brain regions, we found neither effects of the single genotypes nor interaction effects on the VTA or the ventral striatum. At first glance, this is in contrast to the reported animal literature which showed strong evidence for an OT-DA interaction in these mesolimbic structures. However, there are aspects which could be responsible for our negative findings. First, most of the animal research focuses on OT-DA interactions in the context of sexual behavior which probably leads to a stronger activation of the endogenous OT and DA systems than our fMRI task. One could hypothesize that interaction effects in mesolimbic structures like the VTA or the ventral striatum can only be seen in a condition of sexual arousal. Second, the fMRI task we used neither robustly activates the VTA nor the ventral striatum which is a clear drawback for the identification of subtle gene effect. In a future study one should test this hypothesis using an appropriate fMRI task to address these mesolimbic structures.

No additional effect could be found for the fusiform gyrus, either. Therefore, the effect of CD38 genotype on fusiform activation (Sauer et al., 2012) is obviously independent of COMT val158met polymorphism. However, it could still be influenced by other genetic variants. This remains to be investigated in the future.

Regarding amygdala activation, we found no single gene effects but strong evidence for an epistasis effect of both genes reflecting an interaction between the OT and the DA system in the response to socially relevant stimuli. The effect of central OT secretion, as indexed by CD38 rs3796863 genotype, was strongly influenced by the COMT rs4860 genotype (Figures 2, 3). While increased OT secretion (CD38 rs3796863 A+ group) attenuated amygdala responses to socially relevant stimuli under the high DA condition (homozygote COMT rs4860 met allele carriers), it was facilitated under the low DA condition (homozygote COMT rs4860 val allele carriers). Furthermore, since there was no difference between the A+ and the A− group in COMT val158met heterozygotes, the results support the existence of a COMT gene dose effect on the CD38 modulated amygdala activation. Taking it the other way around, the effect of COMT val158met genotype on amygdala activation is strongly depending on central OT secretion. This could explain the heterogeneity of imaging genetics studies on COMT and amygdala activation ranging from increased activation in met allele carriers (e.g., Smolka et al., 2005; Rasch et al., 2010; Lonsdorf et al., 2011) to higher activation in val allele carriers (e.g., Domschke et al., 2008; Lelli-Chiesa et al., 2011). As long as OT system activity and sensitivity is not controlled, sample stratification effects might strongly influence the effect of the COMT genotype. The study by Domschke and colleagues (2008) for example investigated patients with panic disorder. Since anxiety disorders have been associated with oxytocin system alterations (Opacka-Juffry and Mohiyeddini, 2012), it could be speculated that the COMT effects are related to OT system specificities in these patients. However, it has to be emphasized that these relations between anxiety and OT are sex specific (Weisman et al., 2012) but the study by Domschke and colleagues investigated a mixed sample. Another example where the interaction of DA and OT could be important is the study by Lelli-Chiesa and colleagues (2011). They investigated a sample of patients with bipolar disorder and found reduced amygdala activation in met allele carriers. Interestingly, it has been known for a longer time that mood disorders, particularly depression, are associated with OT system alterations (Purba et al., 1996) and it has been shown recently for depressed uni- and bi-polar patients to have reduced OT serum levels (Ozsoy et al., 2009). Therefore, alterations in the patient's OT system could have influenced the COMT effect reported by Lelli-Chiesa and colleagues (2011). Interestingly, there is also a report of a slight association between the COMT met158 allele and teacher rated anxiety in autistic students (Gadow et al., 2009) which nicely fits to our finding of an increased amygdala activation in CD38 risk allele carriers also carrying the COMT met158 allele.

Our results could also be explained in the context of the warrior/worrier model (Goldman et al., 2005). Since homozygote met allele carriers (worriers) are more sensitive to stressors and have a higher trait anxiety (Stein et al., 2005), they might particularly benefit from an increased OT level as present in the A+ carriers of the CD38 gene leading to the well-known stress dampening effect of OT on the amygdala. This might be reflected in the reduced amygdala activation to social stimuli in metmet/A+ individuals compared to metmet/A− individuals. In contrast homozygote val allele carriers (warriors) might be less sensitive to social stimuli as has been shown for faces (Drabant et al., 2006) which might be modulated by OT. In this case, increased OT (CD38 A+ carriers) could enhance the salience of social stimuli or socially relevant aspects of a picture like the eyes (Guastella et al., 2008) which then might increase amygdala response (Gamer and Buchel, 2009) as seen for the valval/A+ carriers compared to the valval/A− carriers.

However, this epistasis effect was exclusively present for the PLA condition while the application of intranasal OT completely diminished the observed interaction (Figure 3). It could be assumed that the presence of exogenous OT eliminates the subtle effects of differential endogenous OT secretion related to the genetic variant in CD38. While CD38 has been found to be relevant for the auto regulation of OT secretion, particularly during the administration of exogenous OT (Lopatina et al., 2010), the differences between CD38 genotypes might be less relevant when exogenous OT strongly influences this pathway. Therefore, since we found no effect of genetic variants after OT application, neither the CD38 nor the COMT genotype seem to substantially impact the pharmacologic effect of OT.

In general, when interpreting the results, we have to take into account that the direct biological pathways underlying are not clear. CD38 is not specific for the OT system and the effect could theoretically be due to other effects of CD38 like its impact on insulin secretion (Kim et al., 2008). However, both the specificity regarding neurotransmitter secretion in the brain (Jin et al., 2007) as well as the fact that the CD38 gene effect observed here was strongly diminished by OT administration supports the assumption that our results are related to the OT specific effect of CD38. Regarding COMT it has to be taken into account, that the enzyme is mainly expressed on cortical sites (Matsumoto et al., 2003) suggesting an indirect influence via a top-down modulation of amygdala regions.

One major shortcoming of our study is the low number of subjects in the extreme groups. This is mainly due to the fact, that group assignment was performed *post-hoc* according to subjects' genotype. Therefore, replication of our results is definitely needed. Nevertheless, our results are an interesting starting point for future research leading toward a better understanding of OT-DA interactions in humans. For example, it would be interesting to see how this epistasis effect reacts to other intentional changes of the OT-DA system, e.g., inhibition of DA signaling via neuroleptic drugs.

Taken together, to our knowledge this is one of the first studies demonstrating an OT × DA interaction in humans. While such interactions have been shown in animal models, mainly in the context of sexual and pair bonding behavior and in regions of the mesolimbic DA system (Baskerville and Douglas, 2008), we could demonstrate that they might also be relevant for a very important basic process underlying human social functioning: the processing of socially relevant stimuli and in a core structure of the limbic system, the amygdala. Although the biological underpinnings of the observed interaction have still to be elucidated, it could be argued that for a substantial understanding of the effect of social hormones on the social brain and human social behavior, interactions with other transmitter systems like the DA system have to be taken into account.

### Conflict of interest statement

The authors declare that the research was conducted in the absence of any commercial or financial relationships that could be construed as a potential conflict of interest.
